# Clinical Features, Survival, and Burden of Toxicities in Survivors More Than One Year After Lung Cancer Immunotherapy

**DOI:** 10.1093/oncolo/oyac140

**Published:** 2022-08-16

**Authors:** Melinda L Hsu, Joseph C Murray, Kevin J Psoter, Jiajia Zhang, Durrant Barasa, Julie R Brahmer, David S Ettinger, Patrick M Forde, Christine L Hann, Vincent K Lam, Benjamin Levy, Kristen A Marrone, Tricia Patel, Valerie Peterson, Sarah Sagorsky, Michelle Turner, Valsamo Anagnostou, Jarushka Naidoo, Josephine L Feliciano

**Affiliations:** Sidney Kimmel Comprehensive Cancer Center at Johns Hopkins University, Baltimore, MD, USA; Bloomberg-Kimmel institute for Cancer Immunotherapy, Baltimore, MD, USA; Sidney Kimmel Comprehensive Cancer Center at Johns Hopkins University, Baltimore, MD, USA; Bloomberg-Kimmel institute for Cancer Immunotherapy, Baltimore, MD, USA; Department of Pediatrics, Johns Hopkins University, Baltimore, MD, USA; Sidney Kimmel Comprehensive Cancer Center at Johns Hopkins University, Baltimore, MD, USA; Bloomberg-Kimmel institute for Cancer Immunotherapy, Baltimore, MD, USA; Sidney Kimmel Comprehensive Cancer Center at Johns Hopkins University, Baltimore, MD, USA; Bloomberg-Kimmel institute for Cancer Immunotherapy, Baltimore, MD, USA; Sidney Kimmel Comprehensive Cancer Center at Johns Hopkins University, Baltimore, MD, USA; Bloomberg-Kimmel institute for Cancer Immunotherapy, Baltimore, MD, USA; Sidney Kimmel Comprehensive Cancer Center at Johns Hopkins University, Baltimore, MD, USA; Bloomberg-Kimmel institute for Cancer Immunotherapy, Baltimore, MD, USA; Sidney Kimmel Comprehensive Cancer Center at Johns Hopkins University, Baltimore, MD, USA; Bloomberg-Kimmel institute for Cancer Immunotherapy, Baltimore, MD, USA; Sidney Kimmel Comprehensive Cancer Center at Johns Hopkins University, Baltimore, MD, USA; Bloomberg-Kimmel institute for Cancer Immunotherapy, Baltimore, MD, USA; Sidney Kimmel Comprehensive Cancer Center at Johns Hopkins University, Baltimore, MD, USA; Bloomberg-Kimmel institute for Cancer Immunotherapy, Baltimore, MD, USA; Sidney Kimmel Comprehensive Cancer Center at Johns Hopkins University, Baltimore, MD, USA; Bloomberg-Kimmel institute for Cancer Immunotherapy, Baltimore, MD, USA; Sidney Kimmel Comprehensive Cancer Center at Johns Hopkins University, Baltimore, MD, USA; Bloomberg-Kimmel institute for Cancer Immunotherapy, Baltimore, MD, USA; Sidney Kimmel Comprehensive Cancer Center at Johns Hopkins University, Baltimore, MD, USA; Bloomberg-Kimmel institute for Cancer Immunotherapy, Baltimore, MD, USA; Sidney Kimmel Comprehensive Cancer Center at Johns Hopkins University, Baltimore, MD, USA; Bloomberg-Kimmel institute for Cancer Immunotherapy, Baltimore, MD, USA; Sidney Kimmel Comprehensive Cancer Center at Johns Hopkins University, Baltimore, MD, USA; Bloomberg-Kimmel institute for Cancer Immunotherapy, Baltimore, MD, USA; Beaumont Hospital Dublin and RCSI University of Health Sciences, Dublin, Ireland; Sidney Kimmel Comprehensive Cancer Center at Johns Hopkins University, Baltimore, MD, USA; Bloomberg-Kimmel institute for Cancer Immunotherapy, Baltimore, MD, USA

**Keywords:** non-small cell lung cancer, survivorship, immune checkpoint inhibitors, immune-related adverse events, toxicities

## Abstract

**Introduction:**

Anti-PD-(L)1 immune checkpoint inhibitors (ICI) improve survival in patients with advanced non-small cell lung cancer (aNSCLC). The clinical features, survival, and burden of toxicities of patients with aNSCLC alive >1 year from ICI initiation are poorly understood.

**Materials and Methods:**

We defined ICI survivors as patients alive >1 year after ICI start and retrospectively reviewed demographics, treatment, and immune-related adverse events (irAEs). Long-term irAEs were defined as ongoing irAEs lasting >1 year; burden of toxicity measures were based on percentage of days a patient experienced toxicity. Using linear and logistic regression, we evaluated association between demographics and disease characteristics with burden of toxicity.

**Results:**

We identified 114 ICI survivors from 317 patients with aNSCLC. Half (52%) experienced an irAE of any grade, and 23.7% developed long-term irAEs. More ICI survivors with irAES in the first year had never smoked (*P = .*018) or received ICIs as frontline therapy (*P = .*015). The burden of toxicity in the first year significantly correlated with the burden of toxicity afterward (*ρ* = 0.72; *P < .*001). No patients with progressive disease had a high burden of toxicity, and they experienced 30.6% fewer days with toxicity than those with stable disease. Increased duration of therapy was associated with higher odds of experiencing toxicity. Half of ICI survivors with irAEs were still receiving treatment for unresolved irAEs at time of death or last follow-up.

**Conclusion:**

Significant proportions of ICI survivors have unresolved long-term toxicities. These data support a growing need to understand long-term toxicity to optimize management of those treated with ICIs.

## Teaser

Immune checkpoint inhibitors (ICIs) have transformed the treatment landscape for patients with advanced non-small cell lung cancer, contributing to a growing population of survivors; however, immune-related adverse events are a concern. This article focuses on the burden of toxicity, bridging the gap in knowledge of the long-term management of immune-related adverse events.

**Implications for Practice**

Survivors of advanced non-small cell lung cancer who have been treated with immune checkpoint inhibitors are living longer than ever. High numbers of these survivors, however, are experiencing long-term, immune-related toxicities which often last after they have completed treatment. Longer time on therapy was associated with increased odds of long-term toxicity, and the burden of toxicity in the first year on therapy correlated with long-term toxicity as well.

## Introduction

The National Cancer Institute defines cancer survivorship as the period of time that begins from the day of a cancer diagnosis until the end of life.^1^ The need for focused care of cancer survivors requires a better understanding of the long-term effects that the diagnosis and the treatments have on patients.^1,2^

Immune checkpoint inhibitors (ICIs) have transformed the treatment landscape for patients with advanced non-small cell lung cancer (aNSCLC),^3-5^ contributing to a rapidly growing population of aNSCLC survivors. Randomized controlled trials demonstrate that 51-69% of such patients survive beyond 1 year of therapy.^6-8^ In these studies, the incidence of immunologic toxicities that occur from ICIs, termed immune-related adverse events (irAEs), ranged from 17% to 34%.^9-12^ Most clinical trials report the overall incidence or emphasize the incidence of highest-grade toxicity. However, toxicities may continue long-term and the characteristics, impact, and management of long-term irAEs have not been assessed or reported. Thus, it is likely that the published toxicity data from clinical trials in advanced NSCLC does not represent the full toxicity experience of patients who receive ICIs, particularly in older patients or those ineligible for clinical trials.^13,14^ The extent of such toxicity, which may be more prevalent than reported in clinical trials, has implications for the quality of life (QOL) and care of survivors.

Given this gap in knowledge, we characterized the demographic, clinical characteristics, PFS, and OS, of patients with aNSCLC treated with ICIs at a tertiary academic cancer center who survived at least 1 year from the initiation of ICI therapy. Within this cohort, we further describe the incidence, spectrum, chronicity, and management the long-term irAE experience. We created novel definitions to describe our findings, as universally accepted definitions do not exist in this emerging field. We also focused on describing the burden of toxicity given the lack of a universally validated metric. We assessed the burden of irAE toxicity as a percentage of days a patient experienced toxicity and explored how clinical and disease related factors are associated with burden of toxicity.

## Materials and Methods

### Study Population

We performed a retrospective study of patients with aNSCLC who started therapy with anti-(PD)-L1 ICIs between November 2009 and February 2019 from a Johns Hopkins IRB-approved database. Patients were included if they were: ≥18 years of age, had histologically confirmed stage III or IV NSCLC, had received ≥1 dose of anti-PD-(L)1 ICI, and were alive ≥1 year from the date of initiation of ICI with administrative censoring in 2/2020. We defined this population as ICI survivors (Supplementary Table S1). Patients who received ICIs only in the neoadjuvant setting were excluded.

### Data Collection

Patient demographics, clinical characteristics, and treatment data were abstracted via electronic medical record chart review. Response to ICIs was determined by RECIST 1.1 criteria^15^ where available, or based on radiology reports and clinical evaluation by the treating oncologist. IrAEs were identified based on either pathologic confirmation, clinical improvement with irAE-based management, or multidisciplinary consensus between a minimum of 2 medical oncologists and the relevant organ specialist, in this rank order. Severity of irAEs were graded by CTCAE v5.0 criteria.^16^ Multi-system irAEs were defined as irAEs occurring in more than one organ system either sequentially or concurrently.^17^ Chronic irAEs were defined as irAEs lasting at least 6 months.^18,19^ Long-term irAEs were defined as ongoing irAEs with a duration of at least 1 year, which survivors were still experiencing >1 year after the start of ICI, at time of death, or last follow-up. Late-onset irAEs were defined as irAEs that developed >1 year after initiation of ICI therapy.^20^ Unresolved irAEs were defined as irAEs which were symptomatic and/or still requiring medical management at time of death or last follow-up. Toxicity duration began from when the toxicity was identified to when it was resolved, as documented in the medical record by a nurse, nurse practitioner, or physician seeing the patient. Toxicity resolution was determined when CTCAE grading reached a score of zero, regardless of whether the survivor was still on medications. The burden of toxicity was defined as the percentage of days that patients experienced an irAE within a given time period, at the 1-year mark from start of immunotherapy and overall in follow-up after starting immunotherapy until death or data censure. We categorized patients as having low burden (LB, <50% of days after treatment start) vs high burden (HB, ≥50% of days after treatment start) of irAE toxicity. Terms as introduced here are summarized in Supplementary Table S1. Overall survival (OS) was defined as the time from ICI initiation to patient death from any cause. Progression-free survival (PFS) was defined as the time from ICI initiation until first radiologic and/or clinical progression as defined by treating oncologist.

### Statistical Analysis

Demographic and clinical characteristics of the overall study population were summarized and compared between those who experienced a current or resolved irAE by the first year following ICI initiation (pos-irAE) and those that did not experience an irAE (neg-irAE) using Mann–Whitney *U* tests for continuous and Chi square or Fisher’s exact tests for categorical variables, respectively. L.B. and H.B. groups were compared in a similar fashion. Median time to onset of first irAE was compared for patients with single to those with multi-system irAEs using the Mann–Whitney *U* test. Pearson correlation was used to assess the relationship between percentage of days with an irAE in the first year with the percentage of days with an irAE post-1 year. Unadjusted linear regression was used to evaluate the association of demographic and clinical characteristics with the percentage of days that patients experienced an irAE. Results of these models are presented as the percent difference in toxicity with corresponding 95% CIs (CI). Logistic regression was then used to evaluate associations of demographic and clinical characteristics with experiencing an irAE at any time during follow-up. Results of these models are presented as odds ratios (OR) and 95% CIs. Secondary outcomes assessed included percentage of total days with an irAE during and after the first year of ICI initiation, percentage of irAE days occurring after the first year of ICI adjusting for number of days in the first year and the proportion of irAE days occurring after the first year. A *P*-value <.05 was considered statistically significant and no adjustments were made for multiple comparisons. All analyses were conducted using STATA 16.1 (StatCorp, College Station, TX) or the R statistical environment.

## Results

### Overall Population Demographics

**Figure 1.**
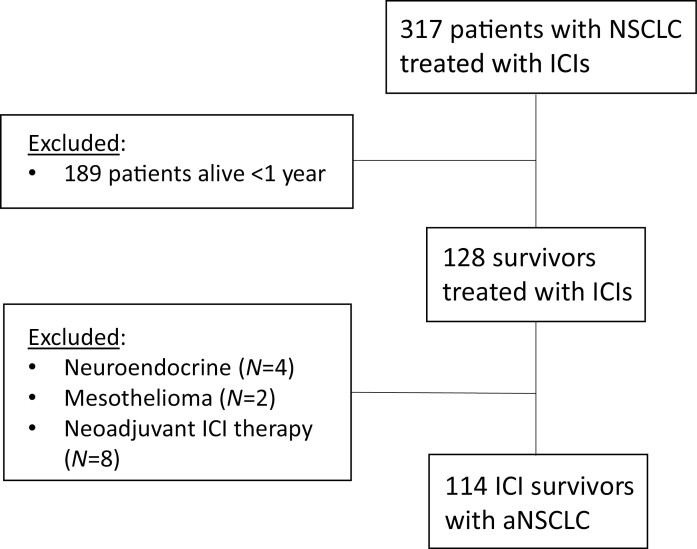
Patients with non-small cell lung cancer treated with immune checkpoint inhibitors at Johns Hopkins University between November 2009 and February /2019. Of 317 aNSCLC patients treated with ICIs, 114 were classified as ICI survivors.

Of 317 aNSCLC patients treated with ICIs, 114 (36%) individuals were alive at one year following ICI initiation and comprised the study population (Fig. 1). These individuals were followed for a median of 26.5 months (range 12.2-106.9 months) to death, last follow-up, or administrative censoring. Approximately half of patients were over 65 years (52.6%) and male (54%), and the majority of patients were White (80.7%), current or former smokers (82.5%), had an ECOG performance status of 0-1 (92.1%) and adenocarcinoma histology (66.7%) (Table 1). Notably, tumor PD-L1 status was not available in 61% of the cohort, as most patients received ICIs prior to 2016 when testing was not an established standard of care or was not required for second-line treatment. Over half of patients in the cohort (51.8%) received ICIs as standard-of-care, in the first (34.2%) or second-line (48.3%) setting or beyond. Eighty-five percent of patients in this cohort demonstrated disease control with stable disease (SD, 43.0%), partial response (PR, 42.1%), or complete response (CR, 6%) as their best response to ICI therapy.

**Table 1.** Demographic and clinical characteristics of individuals with NSCLC receiving ICI therapy overall and by irAE status by 1 year.

	Overall (*n* = 114)	Neg-irAE (*n* = 62)	Pos-irAE (*n* = 52)	*P*-value
Age, years				.171
<65	54 (47.4)	33 (53.2)	21 (40.4)	
65+	60 (52.6)	29 (46.8)	31 (59.6)	
Sex				.629
Male	62 (54.4)	35 (56.5)	27 (51.9)	
Female	52 (45.6)	27 (43.6)	25 (48.1)	
Race				1.000
White	92 (80.7)	50 (80.7)	42 (80.8)	
Black	20 (17.5)	11 (17.7)	9 (17.3)	
Asian	2 (1.8)	1 (1.6)	1 (1.9)	
Race				.987
White	92 (80.7)	50 (80.7)	42 (80.8)	
Non-White	22 (19.3)	12 (19.4)	10 (19.2)	
Smoking status				.018
Current	13 (11.4)	4 (6.5)	9 (17.3)	
Former	81 (71.1)	51 (82.3)	30 (57.7)	
Never	20 (17.5)	7 (11.3)	13 (25.0)	
Marital status				.826
Married	87 (79.8)	50 (82.0)	37 (77.1)	
Single	12 (11.0)	6 (9.8)	6 (12.5)	
Divorced	10 (9.2)	5 (8.2)	5 (10.4)	
ECOG				.857
0	22 (19.3)	12 (19.4)	10 (19.2)	
1	83 (72.8)	46 (74.2)	37 (71.2)	
2+	9 (7.9)	4 (6.5)	5 (9.6)	
Stage at start of IO				.725
III (III, IIIA, and IIIB)	14 (12.3)	7 (11.3)	7 (13.5)	
IV	100 (87.7)	55 (88.7)	45 (86.5)	
Sites of metastasis				
Brain	18 (15.8)	13 (21.0)	5 (9.6)	.125
Liver	17 (14.9)	9 (14.5)	8 (15.4)	.897
Tumor histology				.264
Squamous	30 (26.3)	17 (27.4)	13 (25.0)	
Adenocarcinoma	76 (66.7)	43 (69.4)	33 (63.5)	
Large cell neuroendocrine	3 (2.6)	0 (0.0)	3 (5.8)	
Poorly differentiated carcinoma	3 (2.6)	1 (1.6)	2 (3.9)	
Sarcomatoid	1 (0.9)	1 (1.6)	0 (0)	
Adenosquamous	1 (0.9)	0 (0.0)	1 (1.9)	
Oncogenic driver mutations				
EGFR	4 (4.3)	4 (7.8)	0 (0)	.122
ROS-1	2 (3.9)	2 (7.7)	0 (0)	.255
KRAS	32 (36.8)	23 (50.0)	9 (22.0)	.007
BRAF	4 (5.3)	1 (2.6)	3 (8.3)	.345
RET	2 (4.8)	2 (10.5)	0 (0)	.199
MET	3 (7.3)	0 (0.0)	3 (13.0)	.243
PD-L1 status				.658
Unknown	69 (60.5)	37 (59.7)	32 (61.5)	
0%	13 (11.4)	6 (9.7)	7 (13.5)	
1%-49%	8 (7.0)	4 (6.5)	4 (7.7)	
50+%	24 (21.1)	15 (24.2)	9 (17.3)	
Treatment type				.682
SOC	59 (51.8)	31 (50.0)	28 (53.9)	
Clinical trial	55 (48.3)	31 (50.0)	24 (46.2)	
Number of doses, median (IQR)	13 (19)	12.5 (19)	14.5 (17)	.853
Duration of therapy, median (IQR)	251 (362)	258 (542)	246 (304)	.975
Number of prior systemic therapies				.015
0	39 (34.2)	14 (22.6)	25 (48.1)	
1	55 (48.3)	36 (58.1)	19 (36.5)	
2+	20 (17.5)	12 (19.4)	8 (15.4)	
Prior chemotherapy	75 (65.8)	48 (77.4)	27 (51.9)	.004
Prior targeted therapy	5 (4.4)	5 (8.1)	0 (0)	.062
Prior other systemic therapy	4 (3.5)	2 (3.2)	2 (3.9)	1.000
Number of subsequent systemic therapies				.424
0	55 (48.2)	27 (43.6)	28 (53.9)	
1	23 (20.2)	12 (19.4)	11 (21.2)	
2	24 (21.1)	14 (22.6)	10 (19.2)	
3-9	12 (10.5)	9 (14.5)	3 (5.8)	
Monotherapy				.707
Nivolumab	44 (58.7)	27 (60.0)	17 (56.7)	
Pembrolizumab	21 (28.0)	11 (24.4)	10 (33.3)	
Durvalumab	8 (10.7)	5 (11.1)	3 (10.0)	
MDX-1105	2 (2.7)	2 (4.4)	0 (0)	
Combination therapy				1.000
Dual IO	14 (35.9)	6 (35.3)	8 (36.4)	
IO + chemotherapy	12 (30.8)	5 (29.4)	7 (31.8)	
IO + other	13 (33.3)	6 (35.3)	7 (31.8)	
Best response to IO				.007
Progressive disease	10 (8.8)	10 (16.1)	0 (0)	
Complete response	7 (6.1)	2 (3.2)	5 (9.6)	
Partial response	48 (42.1)	25 (40.3)	23 (44.2)	
Stable disease	49 (43.0)	25 (40.3)	24 (46.2)	

### Incidence of Immune-Related Toxicity

**Figure 2.**
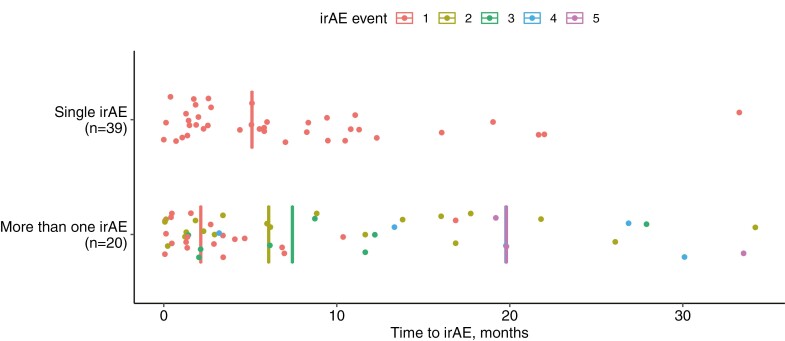
Time to development of immune-related adverse events (irAEs) in ICI survivors. Time to irAE was compared for patients experience a single irAE (top) and more than one, or multisystem, irAE (bottom). Each dot represents the time to irAE from ICI treatment initiation. Median time to irAE events are depicted as vertical lines. Median time to first irAE event was compared by Wilcoxon rank-sum test between patients in the single and multi-system irAE groups (*P* = .03). For multisystem irAEs, the median time to fourth (blue) or fifth (pink) irAEs were the same and are overlapping in the figure.

Immune checkpoint inhibitors survivors from aNSCLC experienced a total of 95 irAEs from ICIs. A total of 59 (51.8%) ICI survivors developed an irAE of any grade by CTCAE 5.0, with the majority being grade 1 (20%) and grade 2 (46%) (Supplementary Table S2). The most common irAEs were pneumonitis (18%), dermatitis (11%), and inflammatory arthritis ((IA), 11%). Single-system irAEs (34.2%) were more commonly observed than multi-system irAEs (17.5%). For those with a single-system irAE, the median time to onset of irAE was 22 weeks, which was significantly longer than the median time of 9 weeks to onset of first irAE for the ICI survivors who experienced multi-system irAEs (*P = .*03, Fig. 2). Forty-three ICI survivors (37.7%) experienced chronic irAEs, most commonly IA (23%), dermatitis (18%), hypothyroidism (16%), and pneumonitis (16%). Almost a quarter (27, 23.7%) developed long-term irAEs, similarly most commonly hypothyroidism (26%), IA (22%), dermatitis (18%), and pneumonitis (18%).

Eighteen ICI survivors (16%) experienced late-onset irAEs, most commonly pneumonitis (44%) and IA (22%). Seven ICI survivors (6%) developed irAEs after discontinuation of ICIs (IA = 3, IA and colitis = 1, colitis = 1, pruritus = 1, aplastic anemia = 1). Pneumonitis was the most common irAE and seen in 21 patients, and in ICI survivors who experienced multi-system irAEs occurred most frequently with dermatitis (*n* = 4), IA (*n* = 3), and sicca syndrome (*n* = 2).

### Immune-Related Toxicity Within First Year of Immunotherapy

Table 1 presents the clinical and treatment-related factors between the neg-irAE and pos-irAE groups (see Methods). There was a higher proportion of never smokers in the pos-irAE (25%) vs neg-irAE (11.3%) group (*P = .*018). A greater proportion of patients in the pos-irAE group received ICIs as their first-line therapy (48%) compared with the neg-irAE group at 1 year (22.6%; *P = .*015). In patients receiving ICIs as second-line therapy or beyond, there was a significantly greater proportion who received previous chemotherapy in the neg-irAE group (77.4%) by the first year, compared with the pos-irAE group (51.9%, *P = .*004). Finally, there was a significant difference in best treatment response to ICIs between the neg-irAE group vs the pos-irAE group. A greater proportion (16.1%) in the neg-irAE group had progressive disease (PD) as the best response to ICI vs zero patients with PD in the pos-irAE group (*P = .*007).

## Burden of Toxicity

### Burden of Toxicity Within First Year of Immunotherapy

We evaluated the burden of toxicity during the first year after starting ICI therapy by comparing low burden (LB) versus high burden (HB) groups (see Methods; Table 2). There were no statistical differences in the distributions of demographic or clinical factors between the LB and HB group, with the exception of best response to therapy. There were significant differences in best response to ICI between LB and HB groups, with 12.4% having PD in the LB group, versus no patients having PD in the HB group (*P = .*035).

**Table 2.** Demographic and clinical characteristics of individuals with NSCLC receiving IO therapy by percentage of days with an immune-related adverse event during first year.

	Low burden (LB) (*n* = 81)	High burden (HB) (*n* = 33)	*P*-value
Age, years			.276
<65	41 (50.6)	13 (39.4)	
65+	40 (49.4)	20 (60.6)	
Sex			.694
Male	45 (55.6)	17 (51.5)	
Female	36 (44.4)	16 (48.5)	
Race			.151
White	69 (85.2)	23 (69.7)	
Black	11 (13.6)	9 (27.3)	
Asian	1 (1.2)	1 (3.0)	
Race			.057
White	69 (85.2)	23 (69.7)	
Non-White	12 (14.8)	10 (30.3)	
Ethnicity			.435
Non-Hispanic	74 (91.4)	32 (97.0)	
Unknown	7 (8.6)	1 (3.0)	
Smoking status			.120
Current	7 (8.6)	6 (18.2)	
Former	62 (76.5)	19 (57.6)	
Never	12 (14.8)	8 (24.2)	
ECOG			.778
0	17 (21.0)	5 (15.2)	
1	58 (71.6)	25 (75.8)	
2+	6 (7.4)	3 (9.1)	
Stage at start of IO			1.000
III (III, IIIA, and IIIB)	10 (12.4)	4 (12.1)	
IV	71 (87.7)	29 (87.9)	
Sites of metastasis			
Brain	16 (19.8)	2 (6.1)	.091
Liver	13 (16.1)	4 (12.1)	.774
Tumor histology			.209
Squamous	18 (22.2)	12 (36.4)	
Adenocarcinoma	58 (71.6)	18 (54.6)	
Large cell neuroendocrine	2 (2.5)	1 (3.0)	
Poorly differentiated carcinoma	1 (1.2)	2 (6.1)	
Sarcomatoid	1 (1.2)	0 (0)	
Adenosquamous	1 (1.2)	0 (0)	
Oncogenic driver mutations			
EGFR	4 (5.8)	0 (0)	.570
ROS-1	2 (5.3)	0 (0)	1.000
KRAS	26 (41.3)	6 (25.0)	.215
BRAF	3 (5.6)	1 (4.8)	1.000
RET	2 (6.7)	0 (0)	1.000
MET	2 (6.9)	1 (8.3)	1.000
PD-L1 status			.631
Unknown	47 (58.0)	22 (66.7)	
0%	10 (12.4)	3 (9.1)	
1%-49%	7 (8.6)	1 (3.0)	
50+%	17 (21.0)	7 (21.2)	
Treatment type			.390
SOC	44 (54.3)	15 (45.5)	
Clinical trial	37 (45.7)	18 (54.6)	
Number of doses, median (IQR)	15 (19)	9 (19)	.696
Duration of therapy, median (IQR)	280 (420)	198 (320)	.510
Number of prior systemic therapies			.432
0	25 (30.9)	14 (42.4)	
1	42 (51.9)	13 (39.4)	
2+	14 (17.3)	6 (18.2)	
Prior chemotherapy	56 (69.1)	19 (57.6)	.238
Prior targeted therapy	5 (6.2)	0 (0)	.319
Prior other systemic therapy	2 (2.5)	2 (6.1)	.578
Number of subsequent systemic therapies			.782
0	39 (48.2)	16 (48.5)	
1	15 (18.5)	8 (24.2)	
2	17 (21.0)	7 (21.2)	
3-9	10 (12.4)	2 (6.1)	
Monotherapy			.923
Nivolumab	33 (60.0)	11 (55.0)	
Pembrolizumab	14 (25.5)	7 (35.0)	
Durvalumab	6 (10.9)	2 (10.0)	
MDX-1105	2 (3.6)	0 (0)	
Combination therapy			.094
Dual IO	8 (30.8)	6 (46.2)	
IO + chemotherapy	11 (42.3)	1 (7.7)	
IO + other	7 (26.9)	6 (46.2)	
Best response to IO			.035
Progressive disease	10 (12.4)	0 (0)	
Complete response	3 (3.7)	4 (12.1)	
Partial response	36 (44.4)	12 (36.4)	
Stable disease	32 (39.5)	17 (51.5)	

### Burden of Toxicity at Any Time in Follow-Up

**Figure 3.**
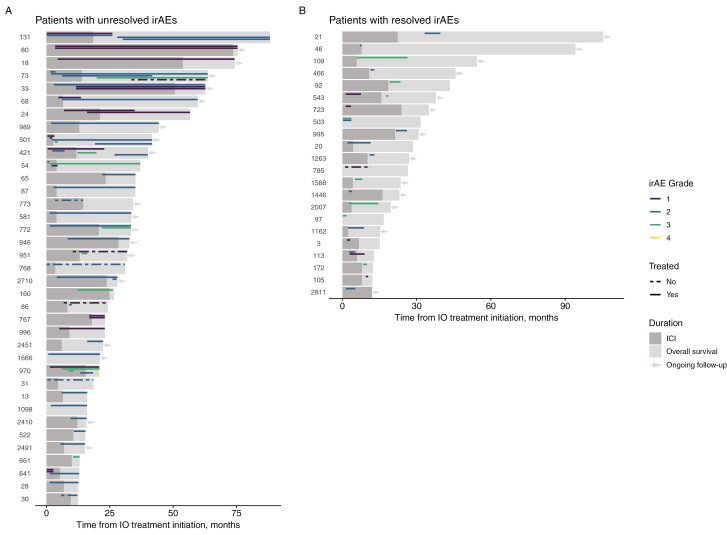
Swimmer plots of ICI survivors stratified by those with unresolved versus resolved irAEs. Each row represents one patient who had unresolved (left) or resolved (right) irAEs during follow-up. The darker gray bars reflect time on immunotherapy and lighter gray bars reflect follow-up or death; arrows at the end of the bars reflect living ICI survivors at the time of data cutoff. Overlying these bars, the duration, grade, and treatment of irAEs are depicted. Grade of the irAE is depicted by color. A dashed line indicates untreated irAEs, and a solid line indicates irAEs that were treated.

We also evaluated the burden of toxicity at any time in follow-up. More than 20% of ICI survivors were classified in the HB group. While >50% of ICI survivors experienced <10% of their days with an irAE, approximately 12% of ICI survivors experienced an irAE for >75% of their time in follow-up after initiating ICI (Supplementary Fig. S1). We also compared the overall burden of toxicity since initiation of ICI between the LB and HB groups (Supplementary Table S3). Similar to burden of toxicity in the first year, there was a numeric difference in the distribution of patients with PD between the LB versus HB groups with 12.2% of patients with PD in the LB group, and no patients with PD in the HB group (*P = .*057). ICI survivors with unresolved irAEs experienced an irAE for a median of 20.0 months (range 0-72 months), which was longer than their median time receiving ICIs of 10.6 months (range 0-73.6 months) (Fig. 3). Patients whose irAEs resolved had a shorter duration of irAE (median 3.0 months, range 0.43-58.2 months) and a shorter duration of ICI therapy than patients with unresolved irAEs (median 8.09 months, range 0-23.9 months). Furthermore, toxicity in the first year of therapy significantly correlated with the burden of toxicity in long-term follow up after one year (*ρ* = 0.72; *P < .*001).

### Associations of Demographic and Clinical Characteristics With Tumor Response

Results of linear regression analyses evaluating the association of patient characteristics with toxicity burden outcomes are presented in Table 3. Compared to those with SD, individuals with PD had 28.7% fewer days with toxicity within the first year (95% CI -52.4, -5%), 32.8% fewer days with toxicity after 1 year of initiating ICI (95% CI −63.1, −2.5%), and 30.6% fewer days of their total days experiencing toxicity (95% CI −55.7, −5.4%). Those with PD were on ICI therapy for a shorter duration of time (median 76.5 days) than those with SD (median 198 days), PR (median 367.5 days), and CR (median 679 days).

**Table 3.** Linear regression evaluating the association of demographic and disease characteristics with outcomes of percentage of days with an irAE.

	Percent days prior to 1 year	Percent days post 1 year	Percent total days	Proportion of total days that were after 1 year
Age, years				
<65	REF	REF	REF	REF
65+	9.4 (−3.67, 22.40)	3.8 (−12.95, 20.53)	7.0 (−6.91, 20.99)	−3.3 (−23.08, 16.50)
Sex				
Male	REF	REF	REF	REF
Female	4.5 (−8.67, 17.64)	9.4 (−7.33, 26.08)	6.1 (−7.86, 20.15)	11.2 (−8.06, 30.55)
Race				
White	REF	REF	REF	REF
Non-White	10.9 (−5.66, 27.36)	2.7 (−18.53, 23.85)	5.8 (−11.92, 23.48)	3.1 (−21.06, 27.27)
Ethnicity				
Non-Hispanic	REF	REF	REF	REF
Unknown	−14.5 (−40.05, 11.07)	−7.8 (−40.54, 24.90)	−9.3 (−36.59, 18.09)	−14.7 (−53.23, 23.80)
Smoking status				
Never	9.9 (−7.27, 27.06)	0.8 (−21.23, 22.76)	8.0 (−10.38, 26.30)	−11.6 (−34.89, 11.66)
Current/former	REF	REF	REF	REF
Marital status				
Married	REF	REF	REF	REF
Single	2.8 (−18.48, 24.11)	19.5 (−7.44, 46.43)	9.9 (−12.84, 32.70)	11.9 (−17.41, 41.29)
Divorced	10.9 (−12.19, 33.99)	14.3 (−14.87, 43.54)	7.6 (−17.06, 32.32)	0.5 (−35.47, 36.52)
ECOG				
0	−3.2 (−20.12, 13.63)	−4.9 (−26.26, 16.39)	−3.9 (−21.83, 14.08)	−5.6 (−31.96, 20.81)
1	REF	REF	REF	REF
2+	−0.2 (−24.92, 24.48)	20.0 (−11.19, 51.22)	7.0 (−19.24, 33.32)	7.0 (−25.70, 39.80)
Stage at start of IO				
III	0.3 (−19.73, 20.29)	−11.1 (−36.55, 14.26)	−5.2 (−26.51, 16.09)	−15.6 (−45.37, 14.25)
IV	REF	REF	REF	REF
Brain metastasis				
No	REF	REF	REF	REF
Yes	−17.3 (−34.98, 0.45)	−4.6 (−27.50, 18.36)	−12.3 (−31.38, 6.73)	10.0 (−22.12, 42.05)
Liver metastasis				
No	REF	REF	REF	REF
Yes	−7.9 (−26.30, 10.45)	−4.4 (−27.87, 19.07)	−8.0 (−27.62, 11.56)	−6.3 (−32.21, 19.56)
PD-L1 status				
0%	REF	REF	REF	REF
1%-49%	−4.3 (−32.34, 23.72)	−4.6 (−44.66, 35.48)	−11.7 (−42.56, 19.07)	−11.5 (−57.18, 34.09)
50+%	−0.2 (−21.65, 21.32)	−4.7 (−35.42, 25.99)	−9.3 (−32.91, 14.32)	−2.3 (−38.50, 33.92)
Treatment type				
SOC	REF	REF	REF	REF
Clinical trial	4.6 (−8.48, 17.74)	7.7 (−8.97, 24.39)	10.1 (−3.78, 23.97)	14.7 (−4.32, 33.82)
Number of doses	0.1 (−0.17, 0.44)	0.4 (0.00, 0.77)	0.3 (0.01, 0.65)	0.6 (0.19, 0.94)
Number of doses received				
1-6	REF	REF	REF	REF
7-13	−2.4 (−20.95, 16.18)	3.4 (−20.16, 27.03)	0.4 (−19.37, 20.17)	11.1 (−16.97, 39.24)
14-25	−7.5 (−25.53, 10.59)	8.1 (−14.86, 31.05)	−0.4 (−19.61, 18.85)	9.9 (−16.61, 36.51)
26+	−1.3 (−20.02, 17.49)	12.6 (−11.22, 36.46)	8.2 (−11.72, 28.21)	30.1 (2.02, 58.22)
Duration	0.0 (−0.2, 0.02)	0.0 (0.00, 0.05)	0.0 (0.00, 0.04)	0.0 (0.02, 0.06)
Duration of therapy, days				
<122	REF	REF	REF	REF
122-251	5.7 (−13.76, 25.11)	10.6 (−14.30, 35.46)	7.6 (−13.44, 28.60)	−19.2 (−49.83, 11.42)
252-483	3.0 (−16.28, 22.28)	20.4 (−4.25, 45.12)	7.7 (−13.1, 28.57)	3.9 (−26.74, 34.51)
484+	−2.1 (−21.51, 17.36)	21.2 (−3.66, 46.11)	11.8 (−9.23, 32.81)	31.2 (0.57, 61.83)
Number of prior systemic therapies				
0	REF	REF	REF	REF
1	−13.3 (−27.87, 1.18)	−12.9 (−31.50, 5.68)	−12.1 (−27.60, 3.45)	13.8 (−7.16, 34.83)
2+	−9.1 (−28.22, 9.95)	−0.5 (−24.91, 23.95)	−1.6 (−22.00, 18.79)	26.2 (−0.09, 52.50)
Number of subsequent systemic therapies				
0	REF	REF	REF	REF
1	−2.7 (−20.2, 14.8)	−15.1 (−37.16, 7.02)	−10.7 (−29.25, 7.93)	−23.5 (−47.33, 0.33)
2	−2.1 (−19.4, 15.1)	−14.1 (−35.89, 7.63)	−5.9 (−24.17, 12.46)	−21.1 (−46.64, 4.42)
3-9	−9.6 (−32.1, 12.9)	−14.1 (−42.47, 14.21)	−9.6 (−33.41, 14.30)	27.2 (−15.56, 69.94)
Monotherapy				
Nivolumab	REF	REF	REF	REF
Pembrolizumab	3.8 (−14.36, 22.05)	−1.7 (−25.14, 21.77)	−2.7 (−22.00, 16.56)	−27.2 (−55.55, 1.22)
Durvalumab	−1.6 (−27.99, 24.76)	−6.0 (−39.95, 28.02)	−27 (−30.71, 25.16)	−16.8 (−62.78, 29.21)
MDX-1105	−22.4 (−72.02, 27.22)	−31.0 (−94.90, 32.97)	−27.1 (−79.64, 25.46)	−
Combination therapy				
Dual IO	REF	REF	REF	REF
IO + chemotherapy	−27.9 (−57.04, 1.14)	−19.0 (−57.06, 19.15)	−21.3 (−53.30, 10.77)	−9.6 (−48.96, 29.84)
IO + other	−7.0 (−35.51, 21.45)	−6.0 (−43.29, 31.32)	−5.1 (−36.44, 26.28)	−9.8 (−49.22, 29.59)
Best response to IO				
Progressive disease	−28.7 (−52.44, −5.03)	−32.8 (−63.06, −2.48)	−30.6 (−55.69, −5.44)	−
Complete response	15.5 (−12.15, 43.06)	22.7 (−12.53, 58.01)	22.2 (−7.06, 51.45)	39.2 (6.65, 71.71)
Partial response	−4.2 (−18.08, 9.67)	0.8 (−16.93, 18.52)	−1.6 (−16.27, 13.13)	16.7 (−2.97, 36.37)
Stable disease	REF	REF	REF	REF

Logistic regression further revealed that patients who had a longer duration of therapy had an increased odds of experiencing a higher percentage of their days with toxicity. Compared to the lowest quartile of duration of therapy (<122 days), patients who received more ICI therapy and categorized in the second (OR = 3.78; 95% CI: 1.14, 12.47), third (OR = 3.49; 95% CI: 1.07, 11.39), and fourth (OR = 3.78; 95% CI: 1.14, 12.47) quartiles all had at least 3 times greater odds of experiencing toxicity (Supplementary Table S4).

### Management of Immune-Related Toxicity in ICI Survivors

The journey for ICI survivors from start of ICI, development/resolution of irAE(s), and treatment for irAE(s) is depicted in Fig. 3. Thirty-seven ICI survivors (32%) had at least one unresolved irAE. The most common unresolved irAEs were hypothyroidism (*n* = 7), IA (*n* = 7), and pneumonitis (*n* = 5). We noted that pneumonitis (*n* = 15), dermatitis (*n* = 10), and colitis (*n* = 6) were the most commonly resolved irAEs. Thirty-one ICI survivors (27%) with unresolved irAEs were still undergoing treatment for their irAE(s) at the time of administrative censoring. A high proportion required continued corticosteroids (45%, 14/31), and 16% (5/31) were receiving additional immunosuppression beyond corticosteroids: methotrexate (*n* = 2) or cyclosporine for (*n* = 1) for IA, mycophenolate for pneumonitis (*n* = 1), and hydroxychloroquine for aplastic anemia (*n* = 1). The spectrum of unresolved irAEs and their maximal management is summarized in Supplementary Fig. S2.

### Survival

The median OS for ICI survivors was 26.5 months (range 12.2-106.9 months), and median PFS was 13 months (range: 0.7-106.9 months). Development of an irAE was not significantly associated with longer median PFS (irAE: 21.2 months vs no irAE: 9.8 months), or OS (irAE: 28.5 months vs no irAE: 24.5 months) in this cohort of NSCLC ICI survivors (Supplementary Fig. S3).

## Discussion

The population of lung cancer survivors with advanced disease is growing rapidly and it is imperative to understand the long-term implications of therapy. In a cohort of patients with aNSCLC treated with ICIs at a single academic institution, we identified a subset of patients who survived more than 1 year after initiation of therapy, which we termed ICI survivors, for whom long-term outcomes and toxicities have previously not been widely reported.

In this study, we identify that half of ICI survivors (52%) experienced an irAE, and almost a quarter (23.7%) developed long-term irAEs. Patients who experienced progressive NSCLC while on ICI therapy were associated with a low likelihood of experiencing long-term irAEs, which is possibly related to the shorter duration of treatment. Toxicity in the first year of therapy strongly correlated with long-term toxicity beyond 1 year. Additionally, we identified that a significant proportion of aNSCLC ICI survivors (32%) had unresolved irAEs at time of death or last follow-up, with 27% receiving ongoing immunosuppressive therapy for these toxicities. Finally, we observed that when one looks exclusively at a population that survived for more than a year, irAEs were not associated with improved PFS or OS.

This is the first study to identify the incidence, ongoing management, and burden of toxicity that patients experience over time from ICI toxicity. Numerous ICI clinical trials have reported longer-term safety data, but with limited information on the ongoing impact of toxicities.^9-12^ Checkmate 017 and Checkmate 057, which compared nivolumab to docetaxel in the second line for aNSCLC, reported overall incident toxicity data and did not discern whether toxicities were attributed to ICI.^21^ Similarly, in the 5-year analysis of Keynote 001, investigators reported an overall incidence of irAEs of 17% in patients who received pembrolizumab in the second-line setting.^9^ Finally, the updated Keynote 024 reported irAEs in 33.8% of patients who received pembrolizumab overall, and in 19.5% of patients who crossed over from chemotherapy to pembrolizumab.^22^ Our analysis focuses solely on patients who survived over a year, rather than all patients who received ICIs, and also evaluated toxicity beyond a year, which is minimally reported in current published data.

Real-world datasets that include patients who were not treated on clinical trials may identify that the incidence of irAEs is much higher than reported in clinical trials.^23^ Our rates of irAEs were similar to a recent real-world cohort of patients with melanoma (*n* = 135), renal cell carcinoma (*n* = 44), and NSCLC (*n* = 38) who survived more than 2 years post ICI (anti-PD-(L)1 or anti-CTLA) start.^24^ In this study, the incidence of acute irAEs was 65%, and chronic irAEs were up to 49%. Although their definitions of acute irAEs (developed during ICI therapy) and chronic irAES (lasting beyond discontinuation of ICIs) differed from ours, their overall toxicity rates are similar to what is reported here. However, the rate of chronic irAEs in their lung cancer cohort was only reported around 18%. The higher irAE rates in our lung cancer-only cohort may reflect our larger cohort size or differences in patients’ demographics including comorbidities. The higher rates seen in retrospective cohorts may be secondary to inclusion of patients with worse performance status or different types of previous therapies than were included in randomized controlled trials. Higher rates of toxicities in our report and other real-world cohorts may also represent differences in attributions of toxicity in clinical trial compared to retrospective analyses.

Higher than expected rates of chronic irAEs were recently reported in a multi-institutional retrospective analysis of chronic toxicity in high-risk, resected melanoma patients who received adjuvant anti-PD-1 therapy.^25^ In this cohort, 43.2% developed chronic irAEs, defined as lasting >12 weeks past discontinuation of ICI therapy, and of those, only 14.4% experienced resolution of their irAE(s). Unlike our analysis, chronic irAEs were associated with superior relapse-free survival compared with acute irAEs. Other emerging analyses suggest that low-grade toxicities may be associated with improved outcomes relative to higher grade toxicity.^26^ It may be important in the future to evaluate the association of toxicity with clinical outcomes such as disease control by differentiating between toxicity grade, type, or duration and all toxicities may not be able to be combined into one group.

These long-term, adverse events we describe likely impact patient functionality and QOL. Some studies have begun to report limited QOL data in immunotherapy clinical trials,^10,27,28^ but only in the first year of survival and may be representative of early QOL changes related to disease response and cancer-driven symptoms. They likely do not capture QOL impacts in the chronic setting from long-term toxicities as they are short-term follow-ups for QOL within a year of initiating therapy.

One method that has been developed to depict the chronicity of adverse events is a measure called the toxicity over time (ToxT) method, which includes temporal information on low-grade toxicity onset, resolution, and patterns over time.^29^ While we cannot perform similar time-dependent statistical modeling for capturing chronic low-grade toxicities in this retrospective analysis, and we did not assess long-term QOL, we surmise that the chronic toxicity we describe here is likely to affect QOL and functionality of survivors. Our findings suggest that patients treated with ICIs may benefit from longitudinal assessment of toxicity over time in order to better understand the long-term impacts of therapies and identify the related needs of these patients.

Immune checkpoint inhibitors survivorship is a new field that demands further study. As such, there are some limitations with our current analyses that we will explore with subsequent evaluation. Standard definitions do not exist for many of the concepts discussed here, including ICI survivors, chronic irAEs, late-onset irAEs, and long-term irAEs. Given the retrospective nature of this study, it is difficult to accurately quantify immune-mediated toxicity, and attribution or resolution of irAEs. We attempted to account for this by 2 independent oncologists adjudicating events, and only including events that were treated with irAE-based management such as corticosteroids or additional immunosuppression. Additionally, inferences are limited as we used unadjusted analyses, and our findings may be confounded by patient characteristics, particularly duration of ICI therapy. Finally, our study population originated from a single academic center and only included patients with aNSCLC who provided consent to have clinical data evaluated retrospectively, which may limit the generalizability of findings. As a future direction, we propose increasing the sample size by expanding the cohort to include survivors of other malignancies commonly treated with immunotherapy such as melanoma or gastrointestinal cancers.

## Conclusion

These data have several important potential clinical applications and provide foundational insights for future studies. Our data can be used as a basis for comprehensive definitions for key, clinically relevant terms currently lacking standardized definitions. This can lead to collaborative prospective understanding of the complexities of identifying ICI toxicity. A better understanding of the risk factors for long-term toxicity may also help to reduce or prevent long-term toxicity in the future and is a next step from this work. Our data support future study to identify the key survivorship needs in patients with aNSCLC who received ICI. Furthermore, because we observed that duration of therapy was associated with burden of toxicity, research to establish the shortest effective duration of ICI therapy that maximizes response while mitigating the risk of long-term toxicity is an important consideration. Finally, future research is also needed to explore the mechanisms underlying both long-term survival and toxicity in ICI survivors, such as genomic or immunologic features that may be prognostic or predictive of both response and toxicity in patients who benefit from immunotherapy.

The findings from our study have important implications for the future of NSCLC and the care of ICI survivors. We provide much-needed evidence to spur the development of tailored long-term assessment of irAEs and their impact on NSCLC ICI survivors, to improve QOL and functionality beyond the benefits of immunotherapy.

## Supplementary Material

oyac140_suppl_Supplementary_Material

## Data Availability

The data underlying this article will be shared on reasonable request to the corresponding author.
