# Supplementary material for: Clinical Features, Survival, and Burden of Toxicities in Survivors More Than One Year After Lung Cancer Immunotherapy
Source: Oncologist. 2022 Aug 16;27(11):971–81. doi: 10.1093/oncolo/oyac140 (PMC9632301; doi:10.1093/oncolo/oyac140)

**Supplemental Material**

Supplemental Table 1. Definitions of terms introduced

| ICI survivor | Patient alive ≥ 1 year from initiation of ICI therapy |
| --- | --- |
| Classification of irAEs |  |
| Chronic irAE | irAE with ≥ 6 months duration |
| Late-onset irAE | irAE developed after 1 year from initiation of ICI therapy |
| Long-term irAE | Ongoing irAE with ≥ 1 year duration and still experienced ≥ 1 year from initiation of ICI therapy |
| Multi-system irAEs | irAEs occurring in more than one organ system either sequentially or concurrently |
| Neg-irAE | No irAE by 1 year from initiation of ICI therapy |
| Pos-irAE | Current or resolved irAE by 1 year from initiation of ICI therapy |
| Unresolved irAE | irAE still requiring medical management at time of death or last follow-up |
| Burden of toxicity | Percentage of days patients experienced an irAE by 1 year, or overall in follow-up |
| High burden (HB) | ≥ 50% of days from ICI initiation |
| Low burden (LB) | < 50% of days from ICI initiation |

Supplemental Table 2. Immune-related adverse events in non-small cell lung cancer immune checkpoint inhibitor survivors by frequency and grade.

| Immune-related Adverse Events | Grade 1  *n*=23 (24) | Grade 2  *n*=53 (56) | Grade 3  *n*=18 (19) | Grade 4  *n*=1 (1) | Total  *n*=95 |
| --- | --- | --- | --- | --- | --- |
| Pneumonitis | 0 | 11 (52) | 10 (48) | 0 | 21 |
| Dermatitis | 4 (31) | 8 (61) | 1 (8) | 0 | 13 |
| Inflammatory arthritis | 3 (25) | 8 (67) | 1 (8) | 0 | 12 |
| Pruritus | 6 (86) | 1 (14) | 0 | 0 | 7 |
| Hypothyroidism | 0 | 7 (100) | 0 | 0 | 7 |
| Colitis | 0 | 6 (100) | 0 | 0 | 6 |
| Diarrhea | 3 (60) | 2 (40) | 0 | 0 | 5 |
| Thyroiditis | 2 (50) | 2 (50) | 0 | 0 | 4 |
| Hypophysitis | 0 | 1 (33) | 2 (67) | 0 | 3 |
| Fatigue | 0 | 3 (100) | 0 | 0 | 3 |
| Hepatitis | 1 (50) | 0 | 1 (50) | 0 | 2 |
| Sicca syndrome | 2 (100) | 0 | 0 | 0 | 2 |
| Pancreatitis | 0 | 0 | 1 (100) | 0 | 1 |
| Nephritis | 0 | 0 | 1 (100) | 0 | 1 |
| DM I | 0 | 0 | 1 (100) | 0 | 1 |
| Anemia | 0 | 1 (100) | 0 | 0 | 1 |
| Aplastic anemia | 0 | 0 | 0 | 1 (100) | 1 |
| Costcochondritis | 0 | 1 (100) | 0 | 0 | 1 |
| Dry eye | 1 (100) | 0 | 0 | 0 | 1 |
| Peripheral neuropathy | 1 (100) | 0 | 0 | 0 | 1 |
| Psoriasis | 0 | 1 (100) | 0 | 0 | 1 |
| Xerostomia | 0 | 1 (100) | 0 | 0 | 1 |

Supplemental Table 3: Demographic and clinical characteristics of individuals with NSCLC receiving IO therapy by percentage of days with an immune-related adverse event during all follow-up.

|  | Low Burden  (n=82) | High Burden  (n= 32) | *P* value |
| --- | --- | --- | --- |
| Age |  |  | 0.629 |
| <65 | 40 (48.8) | 14 (43.8) |  |
| 65+ | 42 (51.2) | 18 (56.3) |  |
| Sex |  |  | 0.314 |
| Male | 47 (57.3) | 15 (46.9) |  |
| Female | 35 (42.7) | 17 (53.1) |  |
| Race |  |  | 0.700 |
| White | 67 (81.7) | 25 (78.1) |  |
| Black | 14 (17.1) | 6 (18.8) |  |
| Asian | 1 (1.2) | 1 (3.1) |  |
| Race |  |  | 0.663 |
| White | 67 (81.7) | 25 (78.1) |  |
| nonWhite | 15 (18.3) | 7 (21.9) |  |
| Ethnicity |  |  | 1.000 |
| nonHispanic | 76 (92.7) | 30 (93.8) |  |
| Unknown | 6 (7.3) | 2 (6.3) |  |
| Smoking status |  |  | 0.832 |
| Never | 14 (17.1) | 6 (18.8) |  |
| Current/former | 68 (82.9) | 26 (81.3) |  |
| Smoking status |  |  | 0.272 |
| Current | 7 (8.5) | 6 (18.8) |  |
| Former | 61 (74.4) | 20 (62.5) |  |
| Never | 14 (17.1) | 6 (18.8) |  |
| Marital status |  |  | 0.729 |
| Married | 65 (81.3) | 22 (75.9) |  |
| Single | 8 (10.0) | 4 (13.8) |  |
| Divorced | 7 (8.75) | 3 (10.3) |  |
| ECOG |  |  | 0.441 |
| 0 | 17 (20.7) | 5 (15.6) |  |
| 1 | 60 (73.2) | 23 (71.9) |  |
| 2+ | 5 (6.1) | 4 (12.5) |  |
| Stage at start of IO |  |  | 0.754 |
| III (III, IIIA and IIIB) | 11 (13.4) | 3 (9.4) |  |
| IV | 71 (86.6) | 29 (90.6) |  |
| Sites of metastasis |  |  |  |
| Brain | 14 (17.1) | 4 (12.50 | 0.776 |
| Liver | 13 (15.9) | 4 (12.5) | 0.776 |
| Tumor histology |  |  | 0.708 |
| Squamous | 21 (25.6) | 9 (28.1) |  |
| Adenocarcinoma | 56 (68.3) | 20 (62.5) |  |
| Large cell neuroendocrine | 2 (2.4) | 1 (3.1) |  |
| Poorly differentiated carcinoma | 1 (1.2) | 2 (6.3) |  |
| Sarcomatoid | 1 (1.2) | 0 (0) |  |
| Adenosquamous | 1 (1.2) | 0 (0) |  |
| Oncogenic driver mutations |  |  |  |
| EGFR | 4 (5.9) | 0 (0) | 0.573 |
| ROS-1 | 2 (5.3) | 0 (0) | 1.000 |
| KRAS | 25 (40.3) | 7 (28.0) | 0.281 |
| BRAF | 3 (5.4) | 1 (5.3) | 1.000 |
| RET | 2 (6.5) | 0 (0) | 1.000 |
| MET | 2 (6.7) | 1 (9.1) | 1.000 |
| PD-L1 status |  |  | 0.705 |
| Unknown | 47 (57.3) | 22 (68.8) |  |
| 0% | 9 (11.0) | 4 (12.5) |  |
| 1-49% | 7 (8.5) | 1 (3.1) |  |
| 50+% | 19 (23.2) | 5 (15.6) |  |
| Treatment type |  |  | 0.137 |
| SOC | 46 (56.1) | 13 (40.6) |  |
| Clinical trial | 36 (43.9) | 19 (59.4) |  |
| Number of doses, median (IQR) | 13 (16) | 18.5 (22.5) | 0.184 |
| Duration of therapy, median (IQR) | 239 (332) | 283.5 (506) | 0.231 |
| Number of prior systemic therapies |  |  | 0.156 |
| 0 | 26 (31.7) | 13 (40.6) |  |
| 1 | 44 (53.7) | 11 (34.4) |  |
| 2+ | 12 (14.6) | 8 (25.0) |  |
| Prior chemotherapy | 56 (68.3) | 19 (59.4) | 0.367 |
| Prior targeted therapy | 5 (6.1) | 0 (0) | 0.320 |
| Prior other systemic therapy | 1 (1.2) | 3 (9.4) | 0.066 |
| Number of subsequent systemic therapies |  |  | 0.784 |
| 0 | 37 (45.1) | 18 (56.3) |  |
| 1 | 18 (22.0) | 5 (15.6) |  |
| 2 | 18 (22.0) | 6 (18.8) |  |
| 3-9 | 9 (11.0) | 3 (9.4) |  |
| Monotherapy |  |  | 1.000 |
| Nivolumab | 32 (57.1) | 12 (63.2) |  |
| Pembrolizumab | 16 (28.6) | 5 (26.3) |  |
| Durvalumab | 6 (10.7) | 2 (10.5) |  |
| MDX-1105 | 2 (3.6) | 0 (0) |  |
| Combination therapy |  |  | 0.366 |
| Dual IO | 8 (30.8) | 6 (46.2) |  |
| IO + chemotherapy | 10 (38.5) | 2 (15.4) |  |
| IO + other | 8 (30.8) | 5 (38.5) |  |
| Best response to IO |  |  | 0.057 |
| Progressive disease | 10 (12.2) | 0 (0) |  |
| Complete response | 3 (3.7) | 4 (12.5) |  |
| Partial response | 35 (42.7) | 13 (40.6) |  |
| Stable disease | 34 (41.5) | 15 (46.9) |  |

Supplemental Table 4: Logistic regression evaluating the association of demographic and disease characteristics with outcomes of percentage of days with an irAE.

|  | >0% of days with irAE prior to 1 year | >0% of days with irAE after to 1 year | >0% of all days with irAE |
| --- | --- | --- | --- |
| Age |  |  |  |
| <65 | REF | REF | REF |
| 65+ | 1.68 (0.80, 3.54) | 1.15 (0.42, 3.16) | 1.75 (0.83, 3.68) |
| Sex |  |  |  |
| Male | REF | REF | REF |
| Female | 1.20 (0.57, 2.52) | 2.11 (0.75, 5.91) | 1.01 (0.48, 2.12) |
| Race |  |  |  |
| White | REF | REF | REF |
| nonWhite | 0.99 (0-.39, 2.52) | 2.50 (0.82, 7.64) | 1.15 (0.45, 2.92) |
| Ethnicity |  |  |  |
| nonHispanic | REF | REF | REF |
| Unknown | 0.70 (0.16, 3.07) | 0.75 (0.09, 6.48) | 0.93 (0.22, 3.90) |
| Smoking status |  |  |  |
| Never | 2.62 (0.96, 7.16) | 0.54 (0.11, 2.57) | 1.94 (0.71, 5.29) |
| Current/former | REF | REF | REF |
| Marital status |  |  |  |
| Married | REF | REF | REF |
| Single | 1.35 (0.40, 4.53) | 1.14 (0.22, 5.80) | 2.14 (0.60, 7.64) |
| Divorced | 1.35 (0.36, 5.01) | 0.63 (0.07, 5.42) | 1.07 (0.29, 3.97) |
| ECOG |  |  |  |
| 0 | 1.04 (0.40, 2.66) | 0.85 (0.22, 3.29) | 0.78 (0.30, 1.99) |
| 1 | REF | REF | REF |
| 2+ | 1.55 (0.39, 6.20) | 1.54 (0.29, 8.25) | 1.86 (0.44, 7.94) |
| Stage at start of IO |  |  |  |
| III | 1.22 (0.40, 3.74) | - | 0.92 (0.30, 2.83) |
| IV | REF | REF | REF |
| Brain metastasis |  |  |  |
| No | REF | REF | REF |
| Yes | 0.40 (0.13, 1.21) | 0.27 (0.03, 2.20) | 0.41 (0.14, 1.17) |
| Liver metastasis |  |  |  |
| No | REF | REF | REF |
| Yes | 1.07 (0.38, 3.01) | 0.68 (0.14, 3.24) | 1.40 (0.49, 3.98) |
| PD-L1 status |  |  |  |
| 0% | REF | REF | REF |
| 1-49% | 0.86 (0.15, 5.00) | - | 0.63 (0.11, 3.71) |
| 50+% | 0.51 (0.13, 2.02) | 0.48 (0.08, 2.79) | 0.38 (0.09, 1.51) |
| Treatment type |  |  |  |
| SOC | REF | REF | REF |
| Clinical trial | 0.86 (0.41, 1.79) | 3.34 (1.10, 10.12) | 1.24 (0.59, 2.59) |
| Number of doses | 1.01 (0.99, 1.02) | 1.01 (0.99, 1.03) | 1.01 (0.99, 1.03) |
| Number of doses received |  |  |  |
| 1-6 | REF | - | REF |
| 7-13 | 0.95 (0.33, 2.71) | 0.58 (0.14, 2.35) | 1.49 (0.53, 4.21) |
| 14-25 | 1.58 (0.58, 4.35) | 1.21 (0.36, 4.10) | 2.08 (0.75, 5.77) |
| 26+ | 1.19 (0.42, 3.39) | REF | 1.62 (0.57, 4.62) |
| Duration of therapy, days | 1.00 (1.00, 1.00) | 1.00 (1.00, 1.00) | 1.00 (1.00, 1.00) |
| Duration of therapy, days |  |  |  |
| <122 | REF | REF | REF |
| 122-251 | 3.27 (0.99, 10.75) | 0.81 (0.05, 13.79) | 3.78 (1.14, 12.47) |
| 252-483 | 3.49 (1.07, 11.39) | 5.74 (0.64, 51.60) | 3.49 (1.07, 11.39) |
| 484+ | 1.57 (0.47, 5.28) | 12.22 (1.43, 104.71) | 3.78 (1.14, 12.47) |
| Number of prior systemic therapies |  |  |  |
| 0 | REF | REF | REF |
| 1 | 0.30 (0.13, 0.70) | 0.26 (0.07, 0.92) | 0.40 (0.17, 0.94) |
| 2+ | 0.37 (0.12, 1.13) | 1.11 (0.32, 3.90) | 0.68 (0.23, 2.05) |
| Number of subsequent systemic therapies |  |  |  |
| 0 | REF | REF | REF |
| 1 | 0.88 (0.33, 2.34) | 0.28 (0.06, 1.34) | 0.67 (0.25, 1.80) |
| 2 | 0.69 (0.26, 1.81) | - | 0.44 (0.17, 1.17) |
| 3-9 | 0.32 (0.08, 1.32) | 0.59 (0.11, 3.00) | 0.21 (0.05, 0.85) |
| Monotherapy |  |  |  |
| Nivolumab | REF | REF | REF |
| Pembrolizumab | 1.44 (0.51, 4.12) | 0.75 (0.18, 3.17) | 0.83 (0.29, 2.35) |
| Durvalumab | 0.95 (0.20, 4.51) | - | 0.55 (0.12, 2.58) |
| MDX-1105 | - | - | - |
| Combination therapy |  |  |  |
| Dual IO | REF | REF | REF |
| IO + chemotherapy | 1.05 (0.22, 5.00) | 0.16 (0.02, 1.67) | 0.78 (0.16, 3.79) |
| IO + other | 0.88 (0.19, 4.00) | 0.15 (0.01, 1.52) | 0.65 (0.14, 3.04) |
| Best response to IO |  |  |  |
| Progressive disease | - | - | - |
| Complete response | 2.60 (0.46, 14.73) | 11.50 (1.72, 76.78) | 6.25 (0.70, 55.84) |
| Partial response | 0.96 (0.43, 2.13) | 5.11 (1.34, 19.48) | 1.59 (0.71, 3.56) |
| Stable disease | REF | REF | REF |

Supplemental Figure 1: Distribution of ICI survivors’ percentage of total days with an irAE at any time in follow-up.


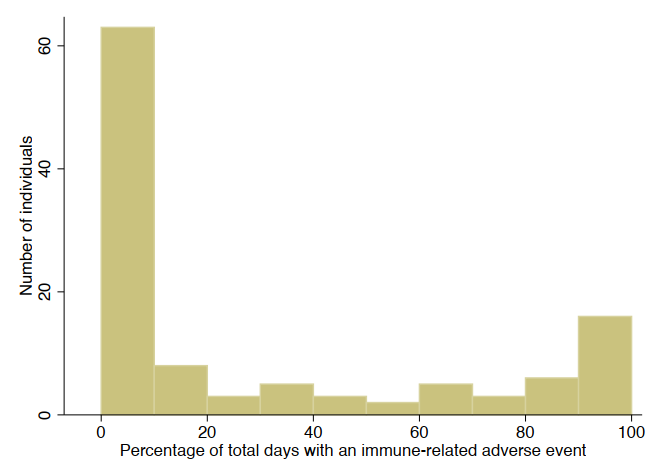


Supplemental Figure 2. Maximal management of ongoing immune-related adverse events in non-small cell lung cancer long-term survivors.


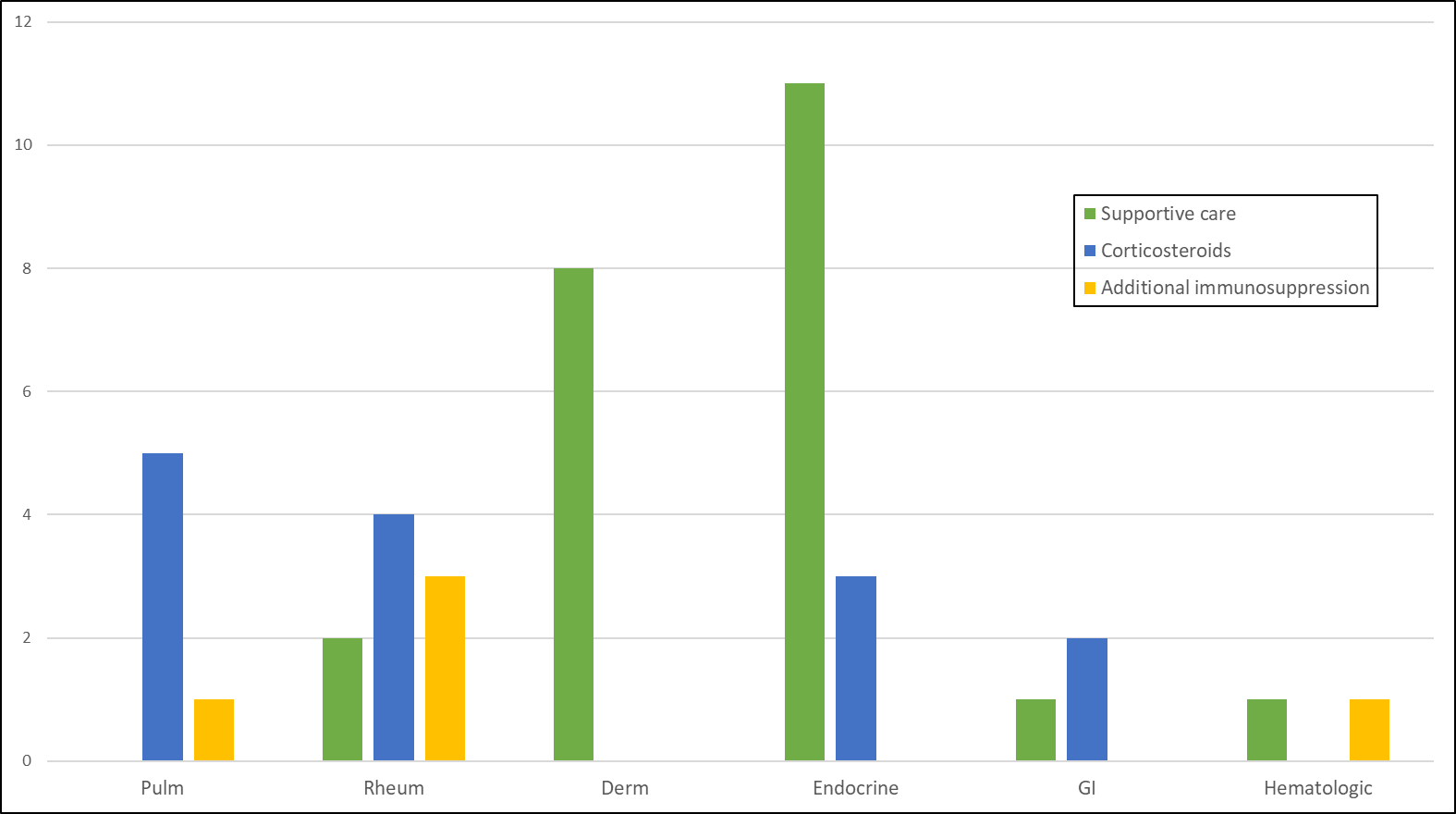


Supplemental Figure 3. Survival Outcomes for non-small cell lung cancer long-term survivors treated with immunotherapy, by development of immune-related adverse event(s)


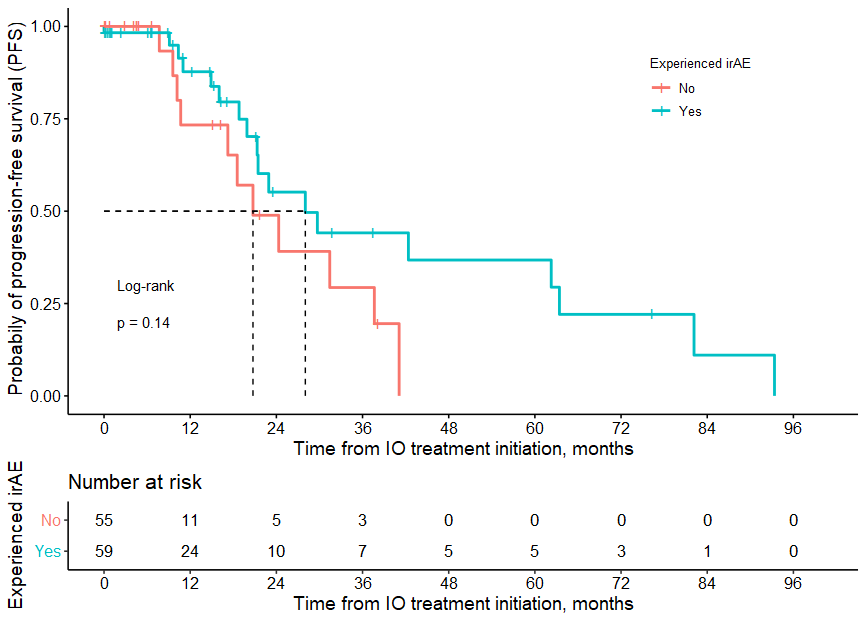


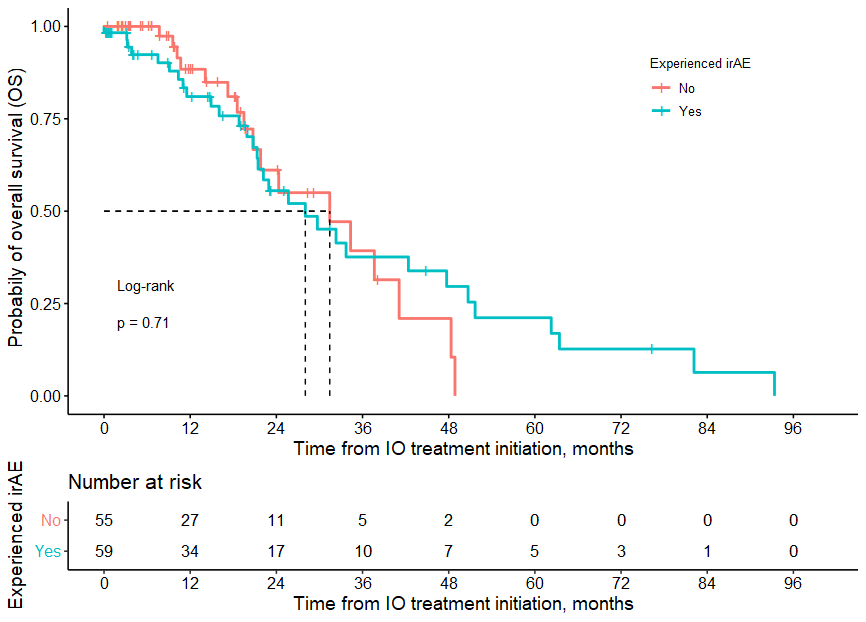

Supplement: oyac140_suppl_Supplementary_Material [file oyac140_suppl_supplementary_material.docx]
